# Almond Grafting for Plum Pox Virus Resistance Triggers Significant Transcriptomic and Epigenetic Shifts in Peaches

**DOI:** 10.3390/ijms26010248

**Published:** 2024-12-30

**Authors:** Julia Corell-Sierra, Régis L. Corrêa, Gustavo G. Gómez, Santiago F. Elena, Juan C. Oliveros, Bernardo Rodamilans, Pedro J. Martínez-García, Pedro Martínez-Gómez, Manuel Rubio

**Affiliations:** 1 Institute for Integrative Systems Biology (I2SysBio), Consejo Superior de Investigaciones Cientificas (CSIC), Universitat de València (UV), 46980 Valencia, Spain; julia.corell@csic.es (J.C.-S.); r.correa@csic.es (R.L.C.); gustavo.gomez@csic.es (G.G.G.); santiago.elena@csic.es (S.F.E.); 2 Department of Genetics, Federal University of Rio de Janeiro (UFRJ), Rio de Janeiro 21941-590, Brazil; 3 The Santa Fe Institute, Santa Fe, NM 87501, USA; 4 Spanish National Center for Biotechnology, CNB-CSIC, 28049 Madrid, Spain; oliveros@cnb.csic.es (J.C.O.); brodamilans@cnb.csic.es (B.R.); 5 Department of Plant Breeding, CEBAS-CSIC, Espinardo, P.O. Box 164, 30100 Murcia, Spain; pjmgarcia@cebas.csic.es (P.J.M.-G.); mrubio@cebas.csic.es (M.R.)

**Keywords:** sharka disease, ‘Garrigues’, chip budding grafting, methylome, WGBS, transcriptome, small RNAs, epigenetic regulation, induced resistance

## Abstract

Sharka disease, caused by the plum pox virus (PPV), negatively impacts stone fruit production, resulting in economic losses. It has been demonstrated that grafting the almond (*Prunus dulcis* (Miller) D.A. Webb) variety ‘Garrigues’ into susceptible peach (*Prunus persica* (L.) Batsch) rootstocks can result in PPV resistance. The molecular circuits related to grafting in *Prunus* species, however, have not been fully investigated. In this study, susceptible peach rootstocks ‘GF305’ were either heterografted with ‘Garrigues’ almond or homografted with the same cultivar. Peach samples were collected at two stages of scion development, with ungrafted plants utilized as controls. Profiles of transcripts, small RNAs (sRNAs), and DNA methylation were obtained and analyzed on a genome-wide scale. Homografting and heterografting significantly altered the transcriptome and methylome of peach rootstocks, with these modifications being more pronounced during the early stages of scion development. The profiles of sRNAs were significantly more impacted when almonds were used as a scion as opposed to peaches, likely due to the transmission of PPV-unrelated viral sequences. Gene expression differences resulting from DNA methylation alterations are more thoroughly documented at the promoter sequences of genes than within their bodies. This study suggests that the ‘Garrigues’ almond variety triggers a complex defense response in the peach rootstock, potentially involving the interplay of epigenetic modifications and small RNA-mediated priming of antiviral defenses, which ultimately may contribute to PPV resistance.

## 1. Introduction

Plum pox virus (PPV; species *Potyvirus plumpoxi*, genus *Potyvirus*, family *Potyviridae*) is the etiological agent of the major disease in stone fruits known as sharka disease [1]. The virus can be non-persistently transmitted by over 20 aphid species or through vegetative means and has been detected in nearly all *Prunus* species. Sharka symptoms are typically linked to a range of economically detrimental traits, including diminished fruit quality and premature fruit abscission [2]. PPV is currently classified as a quarantine virus in many areas due to its significant economic impact [3]. Consequently, identifying sharka resistance strategies has been a primary focus in *Prunus* breeding initiatives.

Unlike the majority of species within its genus, almond plants (*Prunus dulcis* (Miller) D.A. Webb) exhibit notable resistance to PPV [4,5]. It has been observed that grafting the specific almond variety ‘Garrigues’ onto the PPV-susceptible peach (*Prunus persica* (L.) Batsch) cultivar GF305 serves as a natural vaccination against the virus [5]. The symptoms of sharka and the viral load in peaches diminish progressively when grafted with ‘Garrigues’ almonds. Additionally, PPV infection is prevented when almond grafting occurs prior to virus inoculation in peach plants.

Grafting the almond cultivar ‘Garrigues’ onto ‘GF305’ peach (a very susceptible PPV indicator) seedlings heavily infected with PPV-D can progressively reduce disease symptoms and virus accumulation. This response appears to be specific between almond and peach. This induced resistance mechanism has been linked to hormonal changes, primarily involving salicylic acid (SA) pathways, and has been characterized by enhanced transcriptional responses of defense genes. Additionally, grafting ‘Garrigues’ onto ‘GF305’ produced an increase in gibberellic acid and abscisic acid and a decrease jasmonates [6,7]. It was also observed that small RNAs (sRNAs) from three PPV-unrelated viruses in almond scion can migrate to peach rootstocks, suggesting a potential role in the mechanism [8]. The observed protection is unlikely to be a direct consequence of PPV silencing by these mobile viral sRNAs, given the significant sequence divergence among them. They may instead contribute to the priming of peach defenses. Mobile short regulatory RNAs, together with other factors, have been shown to drive changes in mitotically or meiotically heritable epigenetic marks, which can impact gene expression [9]. This raises the possibility that almond sharka’s protective effect on peaches may be also linked to epigenomic alterations.

Epigenetic modifications resulting from chemical modifications to DNA or histones are an integral aspect of the transcriptional regulatory mechanism in living organisms. Both types of epigenetic modifications significantly influence the organization of DNA around histone-associated nucleosomes, hence affecting the accessibility of the RNA transcriptional machinery to specific loci [10]. The dynamic character of these changes makes them key regulators of plant development, as well as adaptive responses to adverse environmental conditions of both biotic and abiotic origin [11]. DNA cytosine methylation marks typically arise at CpG dinucleotides (cytosine–phosphate–guanine sites, where a cytosine is immediately succeeded by a guanine in the DNA sequence). However, in plants, a significant fraction of methylation occurs in non-CpG contexts (CHH, CHG), where H denotes any nucleotide other than guanine. In contrast to symmetrical DNA methylation marks (CpG and CHG), which are often copied during DNA replication, asymmetrical CHH marks are maintained via sRNA-dependent mechanisms [12]. While CpG methylation occurs throughout the genome, CHG and CHH are typically involved in heterochromatin maintenance and suppression of repetitive mobile elements [13].

A prevalent technique for this objective is whole-genome bisulfite sequencing (WGBS), which captures the protection of methylated cytosines from conversion into uracils during bisulfite treatment at a genomic scale [14]. Genome-wide DNA methylation profiles in *Prunus* species have been examined under various conditions and tissues [15,16,17,18,19,20,21,22,23,24]; however, the effect of grafting on DNA methylation in *Prunus* species has not been studied. In addition, DNA methylomes have been extensively investigated across diverse physiological situations in several tissues of hundreds of plant species. Epigenetic marks, including DNA methylation, have been shown to regulate plants’ responses to RNA viruses or viroids [25,26,27,28,29,30,31,32,33]. Epigenetics describes phenomena associated with changes in gene expression that occur without modification to the genomic nucleotide sequence. DNA cytosine methylation is one of the main epigenetic mechanisms in all eukaryotes, and it is produced and maintained over time by several molecular pathways and is likely involved in plant–pathogen interactions, including plant–virus interactions [32,33]. 

In this study, the combined analysis of transcriptome, small RNAome, and methylome profiling were used to examine the circuits involved with peach grafting and their potential roles in explaining the mechanisms of PPV resistance in peach GF305 rootstocks following ‘Garrigues’ grafting. An experimental setup was established in which healthy peaches were homografted with the same cultivar or heterografted with almond. Therefore, the molecular networks of one scion that does not promote PPV resistance (homografting) were compared to another that does, allowing for further exploration of the mechanisms associated with almond sharka protection in plants before viral infection.

## 2. Results

### 2.1. Transcriptomic Responses to Grafting in Peach

**Figure 1:**
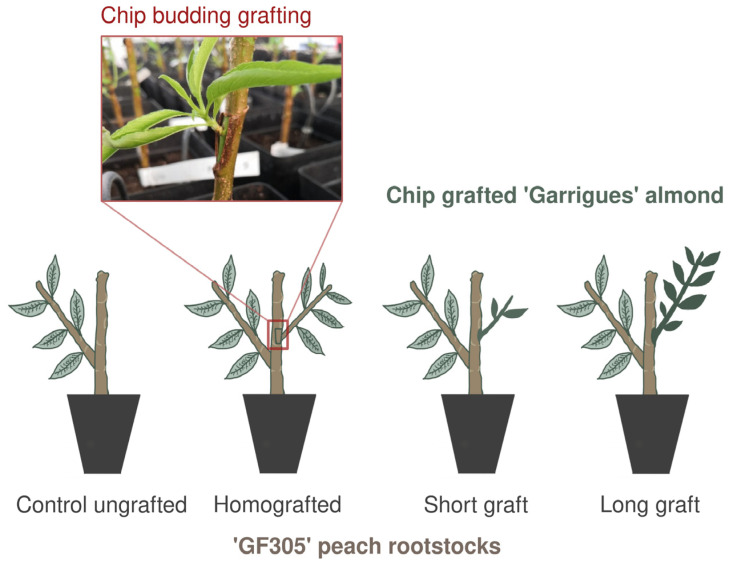
Schematic representation and photograph of the chip-budding grafting technique. Seedlings of peach GF305 were used as rootstocks for grafting with GF305 buds (homografting) and almond cultivar ‘Garrigues’ scions (heterografting). Following initial growth in greenhouse conditions, plants underwent artificial dormancy before scion development resumed. Leaf samples were collected at specific stages: from homografted plants and ungrafted controls after three months, and from heterografted plants with short scions (5–10 cm) after one month and fully developed scions (>20 cm) after three months.

An experimental setup was established using GF305 peach rootstocks, which were homografted with the same peach cultivar or heterografted with the almond cultivar ‘Garrigues’, known for inducing resistance to PPV in peach. The grafted rootstocks were grown under controlled greenhouse conditions, followed by a dormancy period. For the heterografting assay, samples were collected at two stages to compare early-stage almond scions (5–10 cm) with fully developed scions (>20 cm). Ungrafted rootstocks grown under identical conditions were used as controls, as shown in Figure 1.

**Figure 2:**
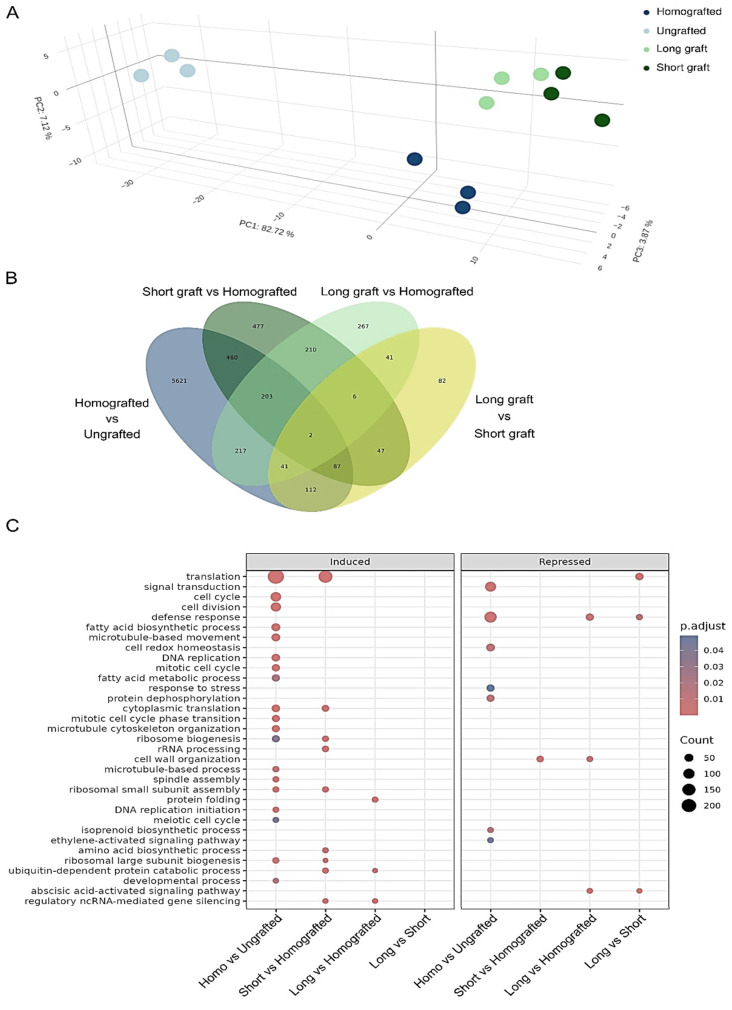
Overview of transcriptomic responses in peach grafted with almond ‘Garrigues’ and homografted peach. (**A**) Principal component analysis (PCA) plot showing sample clustering based on gene expression profiles, with distinct separation between ungrafted controls, homografted samples, and heterografted samples grafted with ‘Garrigues’. (**B**) Venn diagram summarizing the number of differentially expressed genes (DEGs) across each grafting comparison. (**C**) Dot plot of Gene Ontology (GO) enrichment analysis for DEGs, with circle size representing the number of genes in each enriched term, and color heat maps illustrating adjusted *p* values, corrected using the Benjamini–Hochberg method (p.adjust). Full GO enrichment results are available in Supplementary Figure S1.

Principal component analysis (PCA) was performed to explore the overall variance in gene expression profiles captured by Illumina RNA sequencing (mRNA-seq) across experimental groups and assess the separation among samples. The first principal component accounted for 82.71% of the variance, clearly separating the ungrafted controls from the grafted plants. Notably, within the grafted group, PCA differentiated between homografted samples and those heterografted, pointing to distinct expression patterns tied to each grafting approach (Figure 2A).

Differential expression analysis further revealed transcriptional shifts across grafting treatments (Figure 2B, Table S1). When comparing homografted samples with ungrafted controls, 6743 genes with significant changes were observed, reflecting the primary transcriptional impact of homografting. When comparing early-stage almond grafts (with short scions) to homografted samples, 1492 genes were differentially expressed, capturing the initial transcriptional responses to almond grafting. As almond scions developed to maturity, the number of differentially expressed genes (DEGs) diminished, with 987 DEGs in developed almond grafts compared to homografted samples. Furthermore, the transition from early to fully developed almond scions resulted in 418 DEGs, highlighting possible gene expression changes associated with scion maturation.

Gene Ontology (GO) enrichment analysis was conducted on the DEGs (FDR < 0.05) from each comparison (Figure 2C), with the full results for all three aspects available in Supplementary Figure S1. Peach homografting resulted in the highest number of enriched terms, with induced genes primarily associated with translation, cell cycle, cell division, cytoskeleton, and DNA replication, in comparison to control plants. In contrast, the GO terms of repressed genes in homografted samples were related to signal transduction, defense response, cell redox homeostasis, stress response, and protein dephosphorylation.

The comparison between peaches with short almond scions and their homografted counterparts revealed that many GO categories in induced genes overlapped with those identified in homografted plants, along with additional categories associated with rRNA processing, biosynthetic processes, catabolic processes, and regulatory ncRNA-mediated gene silencing. Notably, cell wall organization was one of the few enriched terms among repressed genes in the short scion samples compared to homografted plants (Figure 2C and Figure S1). In the comparison of long almond scions to homografted plants, fewer enriched terms were identified than in the short scion comparison; most were shared with previous comparisons. New terms included protein folding in induced genes and the abscisic acid-activated signaling pathway in repressed genes, the latter also characteristic in the comparison between long and short scions (Figure 2C and Figure S1).

### 2.2. Almond Grafting Impacts Peach Small RNA Profiles

**Figure 3:**
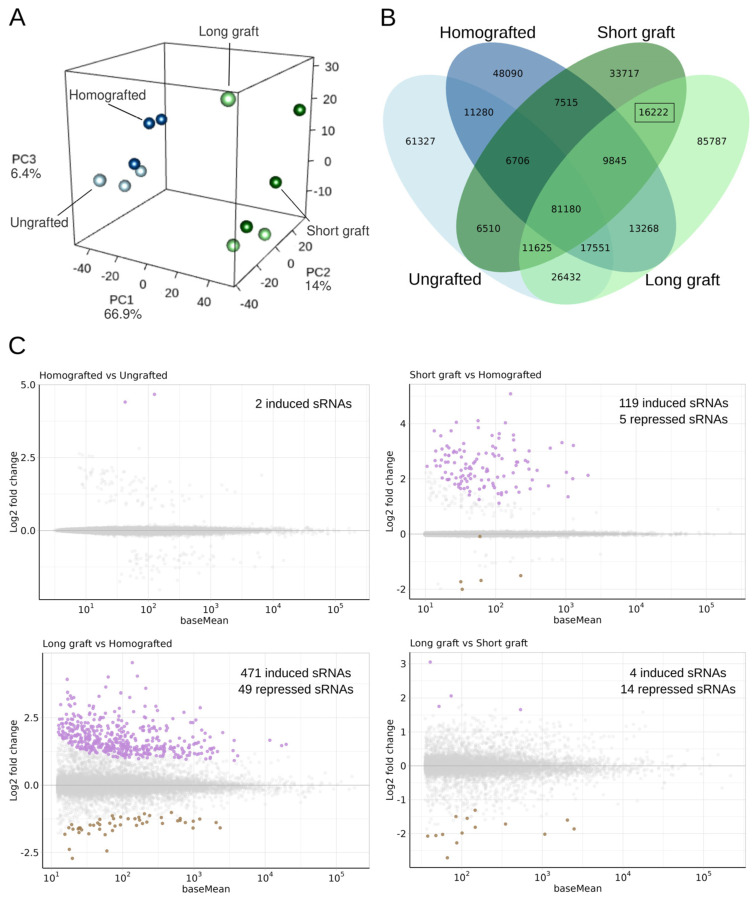
Small RNA (sRNA) profiling across experimental conditions. (**A**) Principal component analysis (PCA) of sRNA expression, with the first principal component (69.9% variance) distinguishing control and homografted samples from those grafted with the almond cultivar ‘Garrigues’. (**B**) Venn diagram showing the intersection of sRNA sequences across all groups. Sequences were defined as characteristic if present in at least two-thirds of the samples per group and ranged from 10 to 24 nucleotides. The diagram illustrates overlapping and unique sequences among the different conditions. (**C**) MA plots showing the differential expression analysis of sRNAs for each comparison: homografted vs. control samples, short scions vs. homografted, long scions *vs.* homografted, and long scions vs. short scions. In each plot, the *x*-axis represents the base mean expression (log_10_ scale), and the *y*-axis indicates the log_2_ fold change (log_2_FC), with significantly differentially expressed sequences highlighted. Induced (in purple) and repressed (in brown) sRNAs were indicated in different colors.

As with the mRNA-seq data, a PCA was conducted to assess overall variance in the small RNA (sRNA) profiles (Figure 3A). Unlike the mRNA-seq results, here the first principal component, which accounted for 69.9% of the explained variance, separated control and homografted samples from those grafted with the ‘Garrigues’ almond cultivar. However, there was no clear separation between samples grafted with short versus long scions at this sRNA level.

To explore these differences further, we defined characteristic sequences as those found in at least two-thirds of the samples in each group, limiting our focus to sequences between 10 and 24 nucleotides (nt). By intersecting these lists, we identified 16,222 unique sequences specific to grafted samples, independent of scion length (Figure 3B). This list was further refined to sequences with an abundance above 0.5 reads per million (RPM) in at least two-thirds of the samples per group and annotated through blastn against the nt database (Figure S2A). Interestingly, of the 1055 sequences queried, 71.75% were annotated as originating from prunus dwarf virus (467/1055), prunus necrotic ringspot virus (278/1055), and peach-associated luteovirus (12/1055). These results are consistent with previous findings in studies of peach grafted with ‘Garrigues’, suggesting a viral signature associated with almond grafting in peach [8].

On the other hand, to examine the expression profile of canonical endogenous sRNAs across conditions, we performed a differential expression analysis on sequences mapping to the peach genome. The mapping percentage for unique sequences (20–24 nt) in each library against the peach genome is shown in Supplementary Figure S2B. Homografted samples showed minimal alteration, with just two sequences differentially expressed compared to controls. In contrast, plants grafted with ‘Garrigues’ exhibited more substantial changes: 124 sRNAs were differentially expressed in peach plants with short almond scions, and 520 sRNAs in those with long scions, both relative to homografted samples. Comparisons between plants with long and short scions revealed fewer differences, with only 18 sequences showing significant expression changes (Figure 3C).

To further characterize the differentially expressed (DE) canonical endogenous sRNAs, we performed an annotation of the sequences. Surprisingly, despite the ribosomal removal step performed using sortmeRNA, a large proportion of sequences were still identified as derived from ribosomal RNA: 531 out of the 564 unique DE sequences across all comparisons. This is likely due to the broader availability of ribosomal RNA sequences of *P. persica* in RNAcentral compared to the default database provided by sortmeRNA. Consequently, only sRNAs not annotated as ribosomal-derived are considered for further discussion. The complete annotation details are provided in Table S2. In homografted plants versus controls, the only identifiable DE sequence corresponded to the microRNA (miRNA) miR-319. In the ‘Garrigues’ grafted plants, which exhibited greater sRNA alterations, 93 of the 551 DE sequences were shared between comparisons. For short almond scions compared to homografted plants, we identified six putative heterochromatic small interfering RNAs (hc-siRNAs) and one tRNA-derived fragment (tsRNA). In long almond scions versus homografted plants, three sequences mapped to phased siRNA loci, and nine sequences were identified as putative hc-siRNAs. Finally, in the comparison between fully developed and short almond scions, one sequence was classified as a putative hc-siRNA. Additionally, two sequences were classified as small fragments of transfer RNAs (tsRNAs), one of which was also ambiguously annotated as an hc-siRNA.

### 2.3. DNA Methylation Landscape of Peach Rootstocks Is Shaped by Almond Scion Grafting

**Figure 4:**
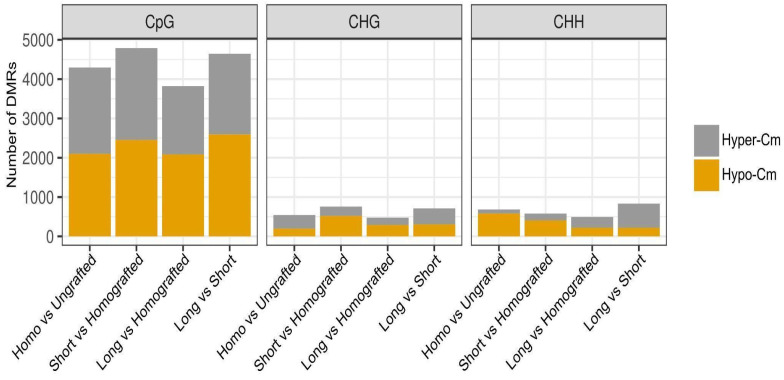
Number of differentially methylated regions (DMRs) in the peach genome under the tested conditions. Hyper- (grey) or hypomethylated (orange) DMRs were analyzed in the three cytosine contexts (CpG, CHG, and CHH). The comparisons performed were homografted (homo) vs. ungrafted plants; peach plants grafted with short or large almond scions vs. homografted plants; and the direct comparison of peach plants with short vs. large almond scions.

To investigate the potential roles of almond scions in modifying DNA methylation patterns of peach rootstocks, methylome libraries (WGBS) were constructed under the previous experimental conditions. Levels of C methylation were analyzed in three contexts: CpG, CHG, and CHH. Differentially methylated regions (DMRs) between conditions were identified in tiles of 100 bases. The majority of the DMRs were found in the CpG context, consistent with their higher prevalence levels (Figure 4, Table S3). Grafting-induced methylation increases (hypermethylation) or decreases (hypomethylation) in peaches to a comparable degree across all comparisons in this context. In contrast to the transcriptome analysis, which indicated that the homografting condition was primarily responsible for the majority of disturbances in the number of DEGs, the effects on the methylomes were consistent in all tested conditions (Figure 4). In accordance with the transcriptome data, the quantity of DMRs was greater in short almond scions compared to larger ones when analyzed against homografted plants (Figure 4). Non-CpG DMRs (CHG/CHH), despite their lower number, exhibit trends comparable to those observed in CpG ones.

A greater number of DMRs was noted in both CHG and CHH contexts in short almond scions compared to the larger ones, relative to homografted plants (Figure 4). GO enrichment analysis was conducted for genes with DMRs located within their annotated sequences across any of the three methylation contexts. Peach homografting resulted in DNA methylation alterations primarily in genes associated with nucleotide metabolism and signal transduction, when compared to control ungrafted plants (Figure S3). Enrichment of genes associated with GO terms related to biotic stress, cytoskeleton, DNA replication, and RNA splicing was also observed. Comparative analysis of peaches with small almond scions and homografted counterparts revealed the presence of several GO categories previously identified in homografted plants, alongside the addition of new categories associated with intracellular organelles, primary and secondary metabolism, as well as transcription and chromatin-related classes (Figure S3). In the comparison of peaches with large almond scions to homografted plants, only GO classes associated with DNA replication were identified (Figure S3).

**Figure 5:**
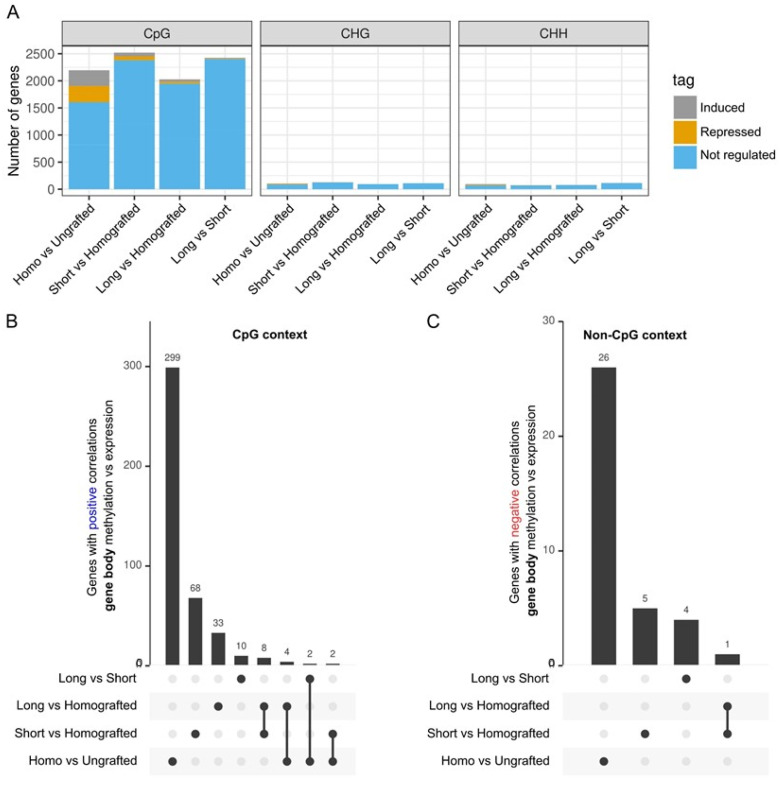
Expression status of genes having differentially methylated regions (DMRs) within their coding sequences under the investigated conditions. (**A**) Genes with DMRs were categorized as induced (grey), repressed (orange), or not differently expressed (blue) in each of the three methylation contexts (CpG, CHG, and CHH) for each specific condition comparison. (**B**) Upset plot showing the number of genes exhibiting a positive correlation between gene body methylation and expression in the CpG context. A positive correlation was defined by genes having either hypomethylation linked to repression or hypermethylation linked to activation. (**C**) Upset plot showing the number of genes exhibiting negative correlation between gene body methylation and expression in the non-CpG context (CHG or CHH). A negative connection was defined by either hypomethylation linked to activation or hypermethylation linked to repression.

We subsequently inquired if the identified DMRs within genes correlated with alterations in expression patterns. Gene body methylation (GbM) at the CpG level may, in certain contexts, correlate with gene activation, while non-CpG (CHG/CHH) marks are often linked to the repression of transposable elements (TEs) or genes [34]. Similar to findings in other plant methylomes, most genes with DMRs were not differentially expressed under any of the tested conditions (Figure 5A). Homografted versus ungrafted peaches had the largest percentage of genes with DMRs that were deregulated among the comparisons made. This phenomenon is probably attributable to the markedly increased quantity of DEGs observed in this condition (Figure 2B, Table S1). However, there were certain instances where the predicted positive or negative associations between methylation levels and differential expression were noted. In the CpG context, instances of hypermethylation associated with gene activation or hypomethylation linked to gene repression were identified. In the non-CpG context, only negative correlations, specifically hypermethylation associated with repression or hypomethylation linked to activation, were taken into account. The peach homografted condition exhibited the highest number of specific genes with a positive correlation between GbM and expression for CpG (299 genes) compared to ungrafted plants (Figure 5B, Table S4). This is followed by the comparison of homografted peaches with those having small (68 genes) or large (33 genes) almond grafts, as well as a direct comparison between plants with small vs. large grafts (10 genes). No GO enrichments were observed for the 299 genes in this control comparison. Analysis of relatives in *Arabidopsis thaliana* revealed that the genes were associated with various pathways, predominantly linked to protein and RNA metabolism and genes related to stress, the cell cycle, chromatin organization, and phytohormones were also included (Figure S4).

In addition to the genes specifically linked to a particular comparison, there were others that were common across different comparisons. The most notable findings in this category are the eight genes shared only by small and large almond scions when compared to homografted plants (Figure 5B). Three of these genes are recognized for their direct involvement in biotic stress responses across various pathosystems: a receptor-like kinase (RLK) is encoded by Prupe.1G542300, a lectin protein kinase family protein by Prupe.4G032900, and a homologue of the *A. thaliana DICER-LIKE 2* (*DCL2*) gene, Prupe.7G047900 (Table S4). The RLK exhibits repression and hypomethylation under both conditions. The lectin protein kinase and *DCL2* are induced and hypermethylated under both conditions (Table S4). As expected, due to their primary function in heterochromatin and the overall limited number of DMRs, only a small number of genes showed negative correlations between GbM levels and transcription in the non-CpG context (Figure 5C). The majority of these genes were linked to ribosomal proteins; however, transcription factors and stress-related genes were also identified (Table S4).

**Figure 6:**
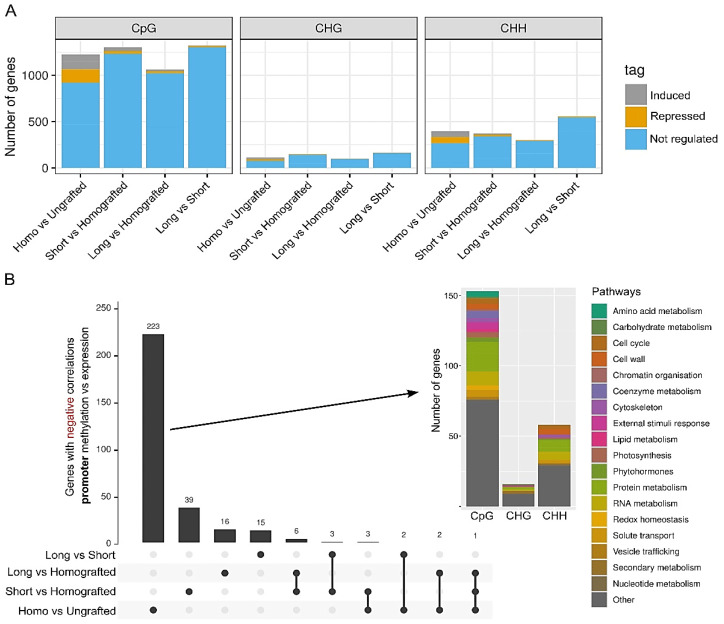
Expression status of genes with differentially methylated regions (DMRs) located near transcriptional start sites (TSS) under the examined conditions. Sequences situated within 2 kb upstream or 200 bp downstream of the gene’s TSS were considered for calculating differences in methylation levels. (**A**) Genes with DMRs were categorized as induced (grey), repressed (orange), or not differently expressed (blue) in each of the three methylation contexts (CpG, CHG, and CHH) for each specific condition comparison. (**B**) Upset plot showing the number of genes exhibiting a negative correlation between gene body methylation and expression. A negative correlation was defined by genes having either hypomethylation linked to transcriptional induction or hypermethylation linked to transcriptional repression. The function of genes specific to the comparison of homografted to ungrafted plants (homo- vs. ungrafted) was examined using MapMan bin pathways (arrow).

Gene expression differences resulting from DNA methylation alterations are more thoroughly documented at the promoter sequences of genes than within their bodies [35]. We examined DMRs located within 2 kb upstream or 200 bp downstream of the transcriptional start sites (TSS) of all peach annotated genes. In the CpG context, the quantity of genes exhibiting DMRs in promoter-proximal regions was approximately half that observed within gene bodies (Figure 6A). In the CHH context, however, the number of DMRs was higher close to TSS than within genes (Figure 6A). This suggests that TEs or repeats situated near promoter regions may have a greater influence on epigenetic differences in the peach genome compared to those located within gene bodies. The observed patterns concerning the number of genes with DMRs near TSS align with findings for intragenic DMRs, indicating that short almond scions exert a greater influence than larger ones when adjusting for homografting effects. The majority of genes with promoter-proximal DMRs across all comparisons were not differentially expressed. Given that promoter methylation is often linked to gene repression for both CpG and non-CpG contexts, genes with negative correlations between TSS-proximal DMRs and expression—that is, those with hypermethylation and repression or hypomethylation with induction—were examined further. The number of genes exhibiting a negative correlation between promoter-proximal methylation and expression was greater in comparisons of homografted plants to ungrafted controls than in comparisons between the two almond scion conditions and homografted plants (Figure 6B, Table S5).

Of the 223 specific genes in this control comparison, the majority of the DMRs were found in the CpG context and encompassed pathways associated with metabolism, chromatin, development, phytohormones, and stress responses (Figure 6B, Table S5). The small almond scion showed additional 39 specific genes with promoter vs. expression negative correlations after the effects of homografted plants were subtracted. These genes included those linked to transcriptional factors (Prupe.1G527100, Prupe.2G204900, Prupe.2G291400, and Prupe.3G174300), phytohormones (Prupe.1G578500, Prupe.2G190100, and Prupe.3G174300), and biotic responses (Prupe.3G144100, Prupe.7G222100, and Prupe.6G054500) (Figure 6B, Table S5). Minimal differences were noted for the large almond scion compared to homografted plants, with a total of 16 genes exhibiting a negative correlation between TSS-proximal methylation and expression. Six genes (Prupe.1G044000, Prupe.1G133500, Prupe.5G120000, Prupe.6G060700, Prupe.8G270600, and Prupe.1G189900) were identified as common to both the short and long graft conditions (Figure 6B, Table S5). Their homologues in *A. thaliana* encode, respectively, a peroxisomal membrane protein, drought-induced 21, a RING/U-box protein, thiaminC, a heat shock protein, and inositol transporter 1. Another gene (Prupe.7G033000) that codes for a disease-resistant LRR family protein was found in the homografted condition as well as in the two almond conditions. In comparison to ungrafted plants, this defense-related gene exhibited significant downregulation (accompanied by TSS-proximal hypermethylation in CpG) as a result of peach homografting. Conversely, a slight induction (along with TSS-proximal hypomethylation in CpG) was noted when grafting peaches with both small and large almond scions when accounting for levels observed in homografted plants (Table S5).

Overall, these results indicate that the grafting process substantially disrupts plant homeostasis, with the effects being exacerbated by the use of Garrigues almonds, particularly during the initial phases.

## 3. Discussion

Grafting experiments have been linked to the interchange of molecules between rootstock and scion in plants, which may result in significant physiological and molecular alterations [36]. This centenary technique has been used in agriculture for a variety of goals, including generating resistance to a number of abiotic and biotic stressors, as well as enhancing production of otherwise susceptible rootstocks [37]. In this regard, it has been demonstrated that grafting ‘Garrigues’ variety almond scions into the sensitive GF305 peach variety confers resistance to the potyvirus PPV [5]. PPV resistance in peaches can be established prior to virus inoculation, suggesting that the mechanism is transferable from almond scions to rootstocks regardless of the presence of PPV in the rootstocks. Given the incomplete understanding of the molecular circuits involved in grafting in peaches, we envisioned an experimental setting to investigate rootstock–scion communication in this plant in greater detail. Uninfected PPV-susceptible peach plants were grafted either with peach (homografting) or with the PPV-inducing resistant almond variety ‘Garrigues’. To assess the potential effects of developing scions, samples were collected after one or three months of growth. The results indicate that grafting procedures in peaches disrupt the plant’s homeostasis at various levels.

Because changes in DNA methylation patterns have been linked to virus infection responses and have been shown to be mediated by grafting in several plants [38], methylomes were examined. Thousands of regions exhibiting differential methylation (DMRs) across the genome were identified between the analyzed conditions (Figure 4, Table S3). Similar magnitudes of effects in the number of DMRs were observed between homografted and ungrafted plants, as well as between almond heterografted and homografted plants. This indicates that heterografting induced more significant alterations in the DNA methylome compared to homografting under our experimental conditions. The number of DMRs was marginally greater for short almond scions compared to long ones, suggesting that the majority of epigenomic changes linked to heterografting take place during the early stages. Furthermore, the analysis of the DMRs across the three C methylation contexts in plants revealed that the majority were in the CpG context (Figure 4). This suggests that grafting influences a greater number of genes compared to transposons, which are typically associated with non-CpG (CHG and CHH) methylation patterns. Peach homografting was linked to DMRs in genes associated with various GO categories, including those related to biotic stress (Figure S3). A significant number of GO classes were identified when comparing short almond scion plants with homografted plants, encompassing both primary and secondary metabolism. This supports previous findings indicating that heterografting can alter plant metabolites [39]. The influence of DMRs located within or near TSS-proximal regions on expression patterns was relatively minor (Figure 5A and Figure 6A). Limited correlation between methylome and transcriptomic data has been observed in various experimental systems in plants [29,40,41,42,43,44,45,46]. This may be linked to various factors, such as context-dependent effects of DNA methylation and the temporal and/or spatial separation between changes in the methylome and transcriptome. Nonetheless, numerous genes exhibited the anticipated correlation between expression and methylation differences within or near promoter-proximal regions, including those associated with stress responses (Figure 5B and Figure 6B, Tables S4 and S5).

Analysis of transcriptome data revealed that peach homografting caused significant transcriptional changes that were less pronounced but comparable in scale to those reported in almond heterografts (Figure 2, Table S1). One limitation of this study is that the plants grafted with short garrigues scion were 2 months younger than the other samples. Compared to the grafting effects, which accounted for 82.71% of the samples’ transcriptional variance, this developmental difference therefore had a very limited impact on the observed expression differences (Figure 2A). In the comparison of homografted plants to control ungrafted ones, induced genes exhibited enrichment in GO classes associated with translation, cell division, and cytoskeleton, whereas repressed genes showed enrichment in signal transduction and stress response categories (Figure 2C). This could suggest that peaches may become more susceptible to stress as a result of homografting. In this line, two sRNAs related to miR319 were also shown to be induced in homografted plants when compared to ungrafted controls (Figure 3C, Table S2). Genes related to this miRNA has been shown to induce susceptibility to infections when overexpressed in other plants [47,48].

To assess the specific effects of almond grafting on peaches, transcriptomic changes induced by short or long ‘Garrigues’ scions were compared with those in homografted plants. Changes in transcript profiles were more pronounced at the early stages of almond grafting into peaches, in accordance with epigenomic data (Figure 2B and Figure 4). Notably, the most significant observation directly associated with the PPV resistance conferred by this almond variety was the statistically relevant induction of genes related to the GO category of ncRNA gene silencing (Figure 2C). The induction of RNA silencing-related genes and the overexpression of sRNAs matching these gene sequences were reported in a previous experiment involving ‘Garrigues’ grafted peaches [8]. In that study, the presence of PPV-unrelated viruses (PNRSV, PDV, and PaLV) was hypothesized as the trigger of this alteration. Similarly, in our results, the majority of DE sRNAs in plants grafted with ‘Garrigues’ were of viral origin (Figure S2B) and corresponded to the same three viruses. This suggests a robust defense response from the peach plant and reinforce the hypothesis that these viruses might play a role in the PPV resistance observed in peach plants grafted with ‘Garrigues’. This process resembles virus meristem exclusion in plants, where RNA silencing responses induced by SA are essential for enhancing virus protection in specific cells [49]. Future research using epigenetic editing tools should investigate whether the presence of the observed expression-inducing CpG marks within the coding region of *DCL2* in almond-grafted peaches can facilitate a sustainable induction of this gene.

## 4. Materials and Methods

### 4.1. Plant Material, Grafting Assays, and Experimental Design

Seedlings of peach ‘GF305’, a well-known rootstock for virus indexing [50], were used as rootstock for chip-budding grafting experiments with our own GF305 (homografting assay) and the traditional Spanish almond cultivar Garrigues (heterografting assay), identified as an inductor of PPV resistance in peach [5,7]. ‘GF305’ rootstocks cultivated during three months in a sealed greenhouse with temperatures between 15 and 30 °C and relative humidity of around 70% were grafted by chip-budding (see detail in the Figure 1) with the ‘GF305’ buds and the ‘Garrigues’ scions. After 1 month of growing in the greenhouse, grafted rootstocks were exposed to a 2-month period in a cold chamber at 7 °C in the dark, to induce artificial dormancy. Afterwards, they were returned to the greenhouse, and peach and almond scions sprouted. In the homografted peach rootstocks, leaf samples were collected after three months in the greenhouse. On the other hand, in the heterografted peach rootstocks, leaves were first collected after 1 month when almond scions were short with around 5–10 cm and after three months when the scions were totally developed with a length of around over 20 cm. Control ungrafted ‘GF305’ rootstocks (sample GF) were included in the experiment as control collecting leave samples after three months in the greenhouse (Figure 1).

### 4.2. RNA-Seq Analysis

Total RNA was extracted using the RNeasy Plant Mini Kit^®^ (Qiagen, Hilden, Germany). The quality and quantity of the total RNA samples were assessed using a NanoDrop^®^ 2000 spectrophotometer (Thermo Fisher Scientific, Wilmington, NC, USA) and diluted at the same concentration (200 ng/L). RNA samples were prepared for library construction and RNA sequencing, assaying three cDNA libraries per treatment. A quality control step was performed for the RNA-Seq (mRNA) reads using FastQC v0.11.9 (https://github.com/s-andrews/FastQC (accessed on 14 July 2023)). Adaptors were trimmed and low-quality bases at the ends of sequences were quality-trimmed to Q20 using bbduk (v38.9) [51]. High-quality mRNA reads were mapped to the reference genome *P. persica* v2.0.a1 (http://www.rosaceae.org/peach/genome, accessed on 20 November 2024) using STAR (v2.7.9a) [52], with default parameters, except for setting --outFilterMultimapNmax to 20. Genomic annotations were obtained from the Genome Database for *Rosaceae* (http://www.rosaceae.org/, accessed on 20 November 2024) in general feature format 3 (GFF3). The gene-level absolute counts were calculated using HTSeq-count (v0.13.5) [53], specifying --order=pos, --stranded=no, --type=gene, --idattr=ID, and --mode=union. Genes with a minimum of five reads in at least two samples were retained for further analysis.

To study the correlation exhibited by the gene expression profiles among the different groups and samples, principal component analysis (PCA) was performed using the prcomp function of the stats R package (v4.2.2), using the matrix of absolute counts normalized by size factor and transformed with the vst function, both functions of the DESeq2 R package (v1.42.1) [54]. The 500 genes with the highest variance were selected for plotting and were graphed using plotly (v4.10.4). Differential expression analysis at the gene level was then computed using DESeq2. Normalization of raw counts was performed using the DESeq2 median of ratios method. Hypothesis testing was performed by a Wald test, and *p*-values were adjusted for multiple testing using the Benjamini–Hochberg procedure. Genes were considered differentially expressed at an adjusted *p*-value threshold of <0.05. The lfcShrink() function in DESeq2 was applied to shrink fold changes.

For each of the comparisons, GO over-representation analysis of the up- and downregulated DEGs was performed using the enricher function of the package ClusterProfiler (v4.10.1) [55]. GO terms were retrieved from Biomart (Ensembl Plants release 60) [56], and terms with a *p*-value < 0.05, adjusted by Benjamini–Hochberg’s correction, we considered significantly enriched.

### 4.3. Small RNA-Seq Analysis

Total RNA was extracted with an RNeasy Plant Mini Kit^®^ (Qiagen, Hilden, Germany), and RNA samples were used for library preparation. The libraries were prepared with TruSeq Small RNA Library Preparation kit and complementary DNA libraries were sequenced with an Illumina GAII machine. Quality control of the small RNA (sRNA) reads was performed using FastQC (v0.11.9) to assess overall read quality, followed by adaptor trimming and quality trimming using fastp (v0.21) [57]. Ribosomal RNA contamination was removed with sortmerna (v4.3.4) [58]. Quantification and removal of sequences containing indeterminate bases were completed with in-house Python scripts. Both absolute and normalized counts (reads per million, RPM) were calculated for each sample, and the individual results were combined to create a count matrix for differential expression analysis and data visualization.

For identifying sequences unique to grafted samples, sequences present in at least two-thirds of the samples within each group were considered characteristic of that group. An additional filter retained only sequences ranging from 20 to 24 nucleotides.

To investigate the correlation patterns in sRNA expression profiles across groups and samples, we conducted principal component analysis (PCA) using the prcomp function in the stats R package (v4.2.2) (https://www.r-project.org/ (accessed on 14 July 2023)). This analysis was based on the absolute count matrix filtered by the criteria outlined above.

Lists of unique sequences were intersected to identify those exclusively present in plants with short or long almond scions. We further filtered this list to include only sequences with an abundance greater than 0.5 RPM in at least two-thirds of the samples per group and then used blastn (v2.14.1) to compare these sequences against the nt database for annotation [59].

For differential expression analysis (DEA), we filtered the original absolute count matrix, retaining only sequences aligned to the *Prunus persica* genome v2.0.a1 (http://www.rosaceae.org/peach/genome (accessed on 20 July 2024)) using Bowtie (v1.3.1) [60]. For each DEA comparison, we further filtered the count table, keeping only sequences with at least five counts in four out of six samples. Normalization of raw counts was performed using the DESeq2 median of ratios method. Hypothesis testing was performed by a Wald test, and p-values were adjusted for multiple testing using the Benjamini–Hochberg procedure. Sequences were considered differentially expressed at an adjusted *p*-value threshold of <0.05. The lfcShrink() function in DESeq2 was applied to shrink fold changes.

The annotation of differentially expressed sequences was performed by mapping with Bowtie (parameters: -v 1 -m 100 -k 1 –best) to *P. persica* miRNA, PHAS loci, and hc-siRNA references from the sRNAanno database [61]. tRNA-derived sequences were identified using the tncRNA-toolkit [62]. Ribosomal sequences of *P. persica* were downloaded from RNAcentral (Release 24).

### 4.4. WGBS-Seq Data

DNA samples were fragmented into 200-400 bp pieces using Covaris S220 focused-ultrasonicator (Woburn, MA, USA). The DNA fragments were repaired, dA-tailed and ligated to sequencing adaptors with all whose cytosines methylated. Then, these fragments were bisulfite-treated with an EZ DNA Methylation Gold Kit (Zymo Research, Irvine, CA, USA). The DNA library was obtained by size selection and PCR amplification. After the construction of the library, the initial quantification was carried out with Qubit 2.0, diluting the library to 1ng/µL. After library quality control, sequencing of different libraries was performed on Illumina HiSeq/NovaSeq platform to obtain 125 bp Paired-End (PE) sequencing. FastQC v0.11.9 was used to assess the quality of the paired WGBS libraries, which were then trimmed with TrimGalore v0.6.6 (https://github.com/FelixKrueger/TrimGalore (accessed on 13 July 2023)), utilizing Cutadapt v3.5 [63] with Python v3.10.4. Paired reads were clipped by 10 nt at their 5’ end, and those with Phred scores lower than 30 or shorter than 36 nt after trimming were discarded. Using Bismark v0.23.1 [64], the remaining reads were PCR deduplicated and aligned to the peach genome v2.0.a1. BAM files were sorted and indexed using samtools v1.15.1 [65].

Methylation calls and differential methylation analysis were conducted using the Methylkit R package version 1.4.1 [66]. Bases exhibiting coverage below 10× and exceeding the 99.9th percentile in each sample were excluded prior to the normalization of mean coverage. Methylation counts were conducted in 100 nt windows, utilizing a tiling step size of 100 nt. Differential methylation statistics among samples were computed using the default χ^2^ test. Differentially methylated regions (DMRs) exhibiting a methylation percentage change of 15 and a *q*-value of 0.05 or lower were selected for analysis.

GO analysis of the hypermethylated and hypomethylated DMRs was conducted utilizing the ClusterProfiler (v4.10.1) package, as previously described for the RNAseq data. The assignment of each DMR to features was conducted using the GenomicRanges v1.52.1 package [67], utilizing the previously described peach annotation file. The assignment of metabolic pathways for the homologous genes of Arabidopsis thaliana was conducted using the Araport11 X4 R1.0 pathway file, which includes MapMan bin mappings.

### 4.5. Code Availability

All code and supplementary files required to reproduce the findings of this study are available in the GitHub repository ncRNA-lab/Garrigues_grafting_omics.

## 5. Conclusions

From a molecular point of view, the whole-genome methylome data generated on peach in this report represent a continued commitment of the *Prunus* community to improve the availability of genetic resources for these economically valuable species. In addition, how epigenetic changes are triggered by grafting is of great interest for researchers and breeders working with fruit trees and some herbaceous crops, because it can help in understanding how a rootstock can control scion phenotype. The obtained results show that homo- and heterografting significantly altered the transcriptome and methylome of peach rootstocks, with these modifications being more pronounced during the early stages of scion development. On the other hand, these epigenetic modifications, among this DNA methylation, represent a potential mechanism that contributes to the regulation of gene expression. The development of the scion did not greatly affect DNA methylation patterns. These comparative methylome studies in grafted and ungrafted materials may contribute to increase knowledge about DNA methylation variants. From an applied point of view, the profiles of sRNAs were significantly more impacted when almonds were used as a scion as opposed to peaches, likely due to the transmission of PPV-unrelated viral sequences. This study suggests that the ‘Garrigues’ almond variety triggers a complex defense response in the peach rootstock, potentially involving the interplay of epigenetic modifications and small RNA-mediated priming of antiviral defenses, which ultimately may contribute to PPV resistance. ‘Garrigues’ almond grafting could thus be used as a natural vaccine against PPV in peach. This response appears to be specific between almond and peach.

## Data Availability

The datasets generated can also be found as a Bio project entitled “Homo and heterografting greatly affects DNA methylation in peach rootstocks” in the NCBI SRA repository with the accession number PRJNA765525 (https://www.ncbi.nlm.nih.gov/bioproject/PRJNA765525, accessed on 7 November 2024).

## Supplementary Materials

The following supporting information can be downloaded at: https://www.mdpi.com/article/10.3390/ijms26010248/s1.
