# Supplementary material for: Almond Grafting for Plum Pox Virus Resistance Triggers Significant Transcriptomic and Epigenetic Shifts in Peaches
Source: Int J Mol Sci. 2024 Dec 30;26(1):248. doi: 10.3390/ijms26010248 (PMC11720244; doi:10.3390/ijms26010248)
Supplement: Supplementary file 1 [file ijms-26-00248-s001.zip › ijms-335849R1-Supplementary Figures S1-S4.pdf]

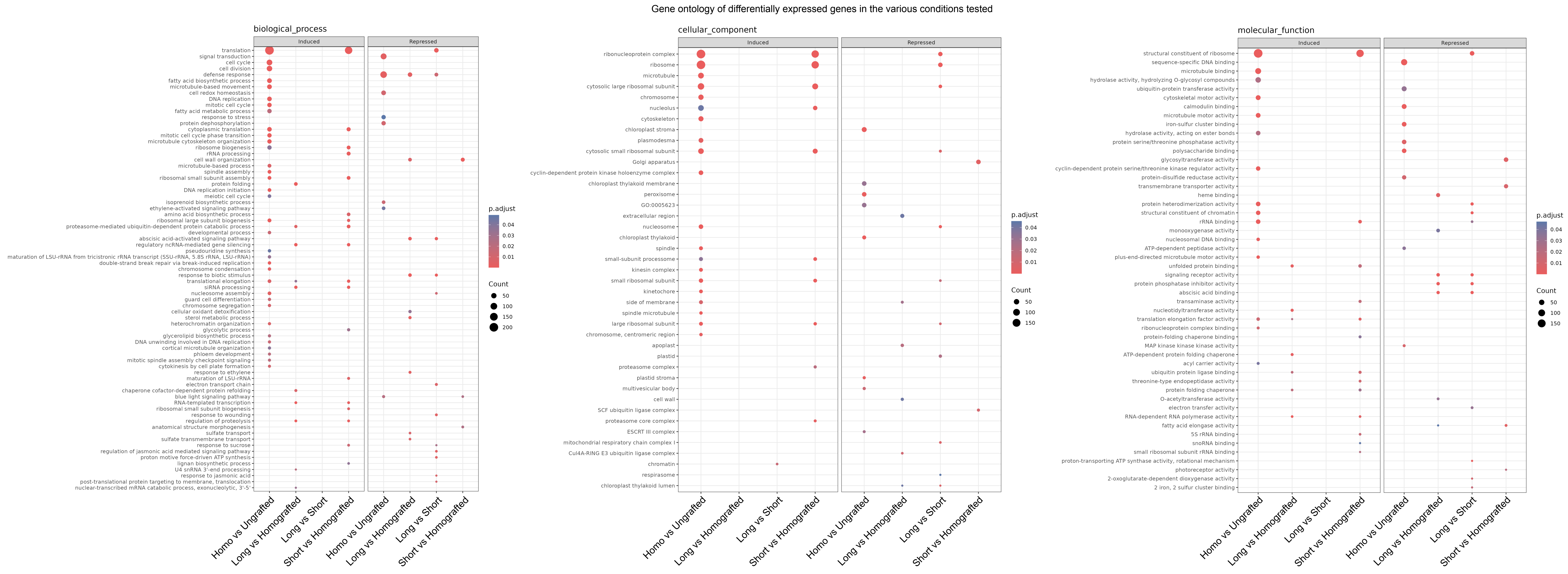
Gene ontology of differentially expressed genes in the various conditions tested
biological_process
Induced
Repressed
translation
signal transduction
cell cycle
cell division
defense response
fatty acid biosynthetic process
microtubule-based movement
cell redox homeostasis
DNA replication
mitotic cell cycle
fatty acid metabolic process
response to stress
protein dephosphorylation
cytoplasmic translation
mitotic cell cycle phase transition
microtubule cytoskeleton organization
ribosome biogenesis
rRNA processing
cell wall organization
microtubule-based process
spindle assembly
ribosomal small subunit assembly
protein folding
DNA replication initiation
meiotic cell cycle
isoprenoid biosynthetic process
ethylene-activated signaling pathway
amino acid biosynthetic process
ribosomal large subunit biogenesis
proteasome-mediated ubiquitin-dependent protein catabolic process
developmental process
abscisic acid-activated signaling pathway
regulatory ncRNA-mediated gene silencing
pseudouridine synthesis
maturation of LSU-rRNA from tricistronic rRNA transcript (SSU-rRNA, 5.8S rRNA, LSU-rRNA)
double-strand break repair via break-induced replication
chromosome condensation
response to biotic stimulus
translational elongation
siRNA processing
nucleosome assembly
guard cell differentiation
chromosome segregation
cellular oxidant detoxification
sterol metabolic process
heterochromatin organization
glycolytic process
glycerolipid biosynthetic process
DNA unwinding involved in DNA replication
cortical microtubule organization
phloem development
mitotic spindle assembly checkpoint signaling
cytokinesis by cell plate formation
response to ethylene
maturation of LSU-rRNA
electron transport chain
chaperone cofactor-dependent protein refolding
blue light signaling pathway
RNA-templated transcription
ribosomal small subunit biogenesis
response to wounding
regulation of proteolysis
anatomical structure morphogenesis
sulfate transport
sulfate transmembrane transport
response to sucrose
regulation of jasmonic acid mediated signaling pathway
proton motive force-driven ATP synthesis
lignan biosynthetic process
U4 snRNA 3'-end processing
response to jasmonic acid
post-translational protein targeting to membrane, translocation
nuclear-transcribed mRNA catabolic process, exonucleolytic, 3'-5'
Homo vs Ungrafted
Long vs Homografted
Long vs Short
Short vs Homografted
p.adjust
0.04
0.03
0.02
0.01
Count
50
100
150
200
cellular_component
ribonucleoprotein complex
ribosome
microtubule
cytosolic large ribosomal subunit
chromosome
nucleolus
cytoskeleton
chloroplast stroma
plasmodesma
cytosolic small ribosomal subunit
Golgi apparatus
cyclin-dependent protein kinase holoenzyme complex
chloroplast thylakoid membrane
peroxisome
GO:0005623
extracellular region
nucleosome
chloroplast thylakoid
spindle
small-subunit processome
kinesin complex
small ribosomal subunit
kinetochore
side of membrane
spindle microtubule
large ribosomal subunit
chromosome, centromeric region
apoplast
plastid
proteasome complex
plastid stroma
multivesicular body
cell wall
SCF ubiquitin ligase complex
proteasome core complex
ESCRT III complex
mitochondrial respiratory chain complex I
Cul4A-RING E3 ubiquitin ligase complex
chromatin
respirasome
chloroplast thylakoid lumen
molecular_function
structural constituent of ribosome
sequence-specific DNA binding
microtubule binding
hydrolase activity, hydrolyzing O-glycosyl compounds
ubiquitin-protein transferase activity
cytoskeletal motor activity
calmodulin binding
microtubule motor activity
iron-sulfur cluster binding
hydrolase activity, acting on ester bonds
protein serine/threonine phosphatase activity
polysaccharide binding
glycosyltransferase activity
cyclin-dependent protein serine/threonine kinase regulator activity
protein-disulfide reductase activity
transmembrane transporter activity
heme binding
protein heterodimerization activity
structural constituent of chromatin
rRNA binding
monooxygenase activity
nucleosomal DNA binding
ATP-dependent peptidase activity
plus-end-directed microtubule motor activity
unfolded protein binding
signaling receptor activity
protein phosphatase inhibitor activity
abscisic acid binding
transaminase activity
nucleotidyltransferase activity
translation elongation factor activity
ribonucleoprotein complex binding
protein-folding chaperone binding
MAP kinase kinase activity
ATP-dependent protein folding chaperone
acyl carrier activity
ubiquitin protein ligase binding
threonine-type endopeptidase activity
protein folding chaperone
O-acetyltransferase activity
electron transfer activity
RNA-dependent RNA polymerase activity
fatty acid elongase activity
5S rRNA binding
snoRNA binding
small ribosomal subunit rRNA binding
proton-transporting ATP synthase activity, rotational mechanism
photoreceptor activity
2-oxoglutarate-dependent dioxygenase activity
2 iron, 2 sulfur cluster binding

A

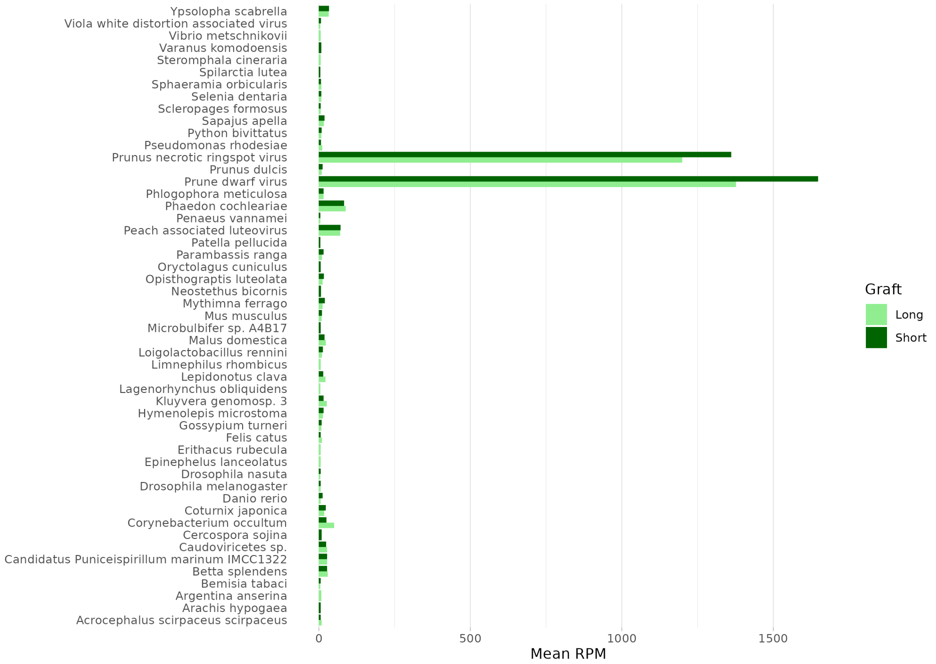

B

Sample	Replicate	Mapped (%)
Ungrafted	1	41,92
Ungrafted	2	47,65
Ungrafted	3	47,85
Homografted	1	60,52
Homografted	2	56,82
Homografted	3	62,02
Long graft	1	42,16
Long graft	2	47,87
Long graft	3	45
Short graft	1	56,99
Short graft	2	54,59
Short graft	3	50,87

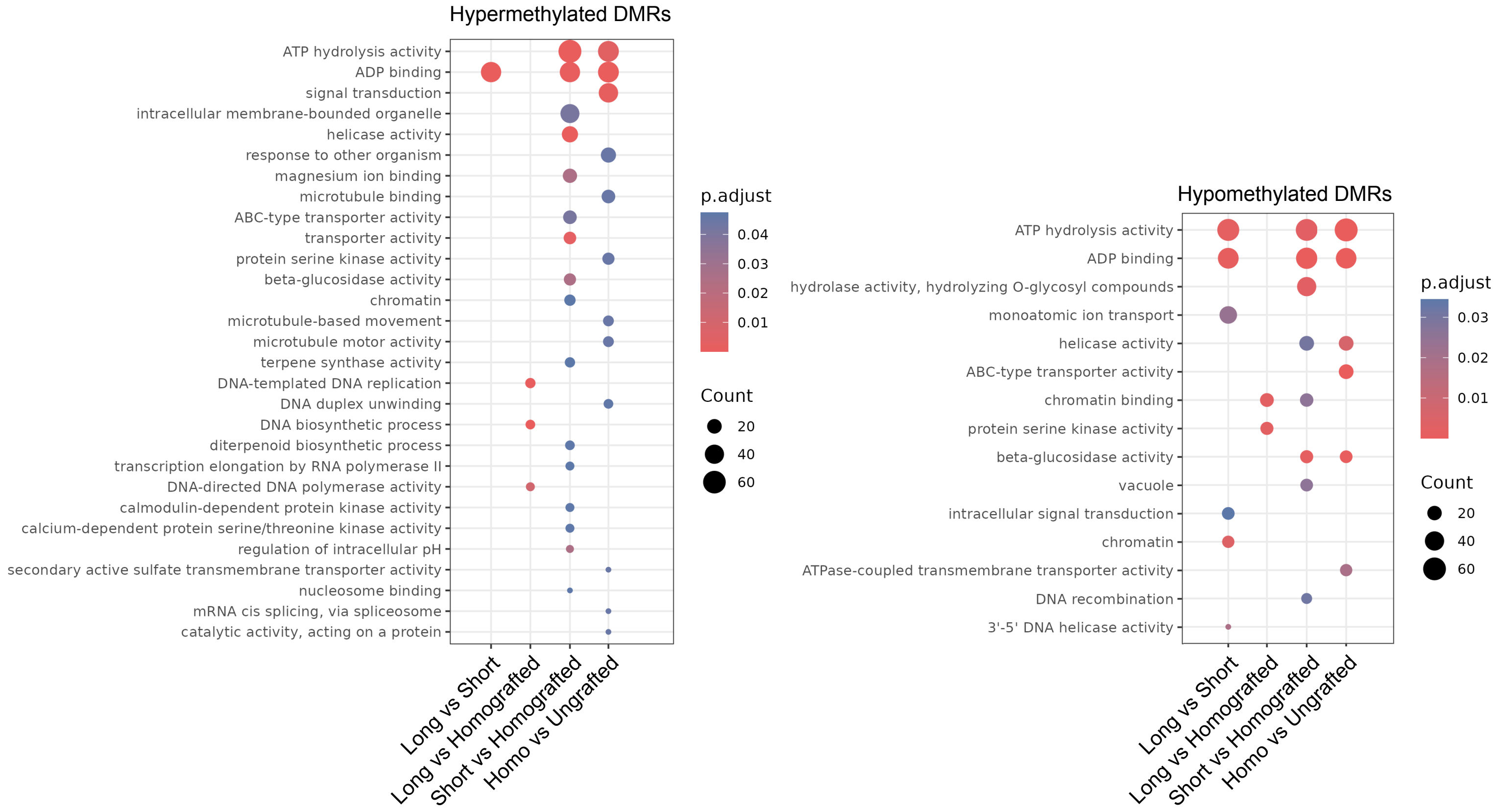
Hypermethylated DMRs
ATP hydrolysis activity
ADP binding
signal transduction
intracellular membrane-bounded organelle
helicase activity
response to other organism
magnesium ion binding
microtubule binding
ABC-type transporter activity
transporter activity
protein serine kinase activity
beta-glucosidase activity
chromatin
microtubule-based movement
microtubule motor activity
terpene synthase activity
DNA-templated DNA replication
DNA duplex unwinding
DNA biosynthetic process
diterpenoid biosynthetic process
transcription elongation by RNA polymerase II
DNA-directed DNA polymerase activity
calmodulin-dependent protein kinase activity
calcium-dependent protein serine/threonine kinase activity
regulation of intracellular pH
secondary active sulfate transmembrane transporter activity
nucleosome binding
mRNA cis splicing, via spliceosome
catalytic activity, acting on a protein
Long vs Short
Long vs Homografted
Short vs Homografted
Homo vs Ungrafted
p.adjust
0.04
0.03
0.02
0.01
Count
20
40
60
Hypomethylated DMRs
ATP hydrolysis activity
ADP binding
hydrolase activity, hydrolyzing O-glycosyl compounds
monoatomic ion transport
helicase activity
ABC-type transporter activity
chromatin binding
protein serine kinase activity
beta-glucosidase activity
vacuole
intracellular signal transduction
chromatin
ATPase-coupled transmembrane transporter activity
DNA recombination
3'-5' DNA helicase activity
Long vs Short
Long vs Homografted
Short vs Homografted
Homo vs Ungrafted
p.adjust
0.03
0.02
0.01
Count
20
40
60

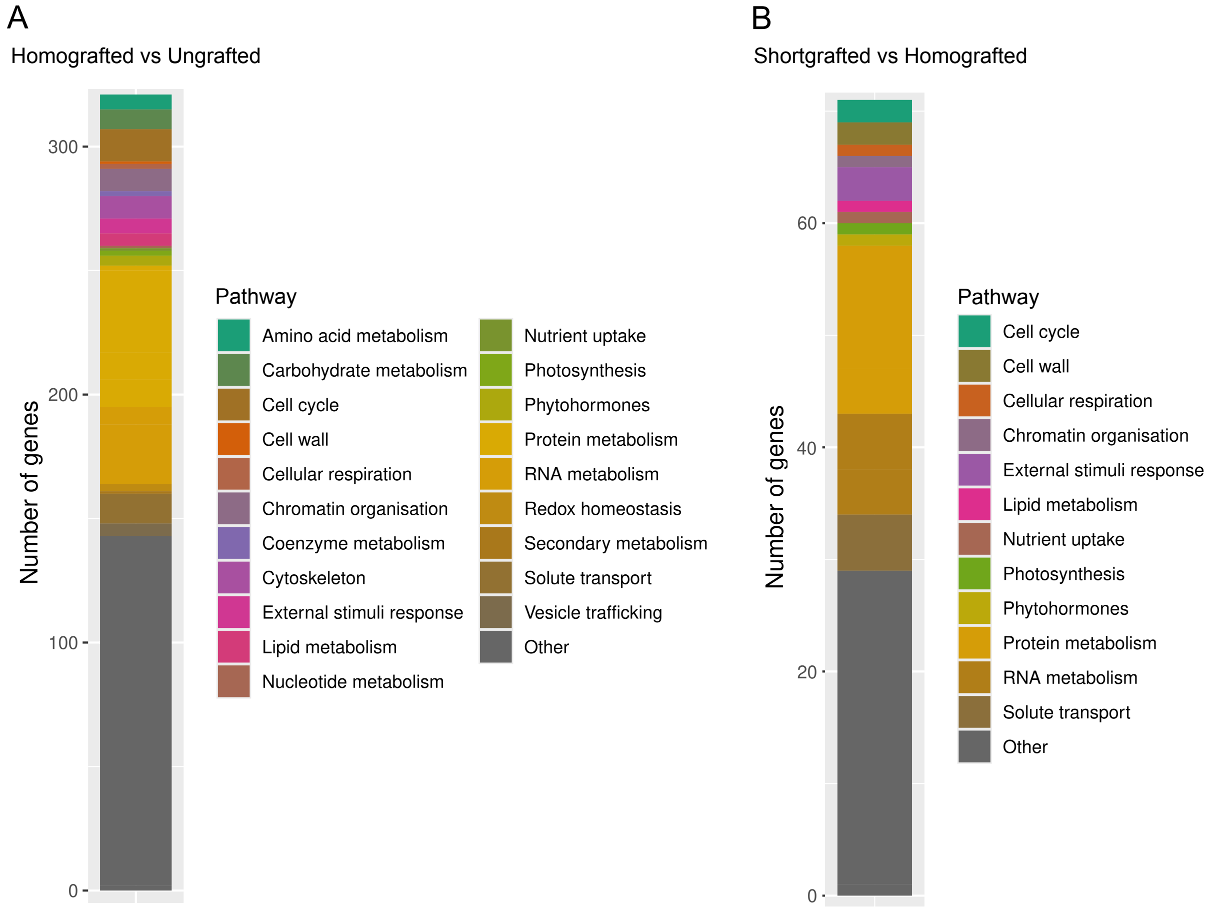
A
Homografted vs Ungrafted
Number of genes
0
100
200
300
Pathway
Amino acid metabolism
Carbohydrate metabolism
Cell cycle
Cell wall
Cellular respiration
Chromatin organisation
Coenzyme metabolism
Cytoskeleton
External stimuli response
Lipid metabolism
Nucleotide metabolism
Nutrient uptake
Photosynthesis
Phytohormones
Protein metabolism
RNA metabolism
Redox homeostasis
Secondary metabolism
Solute transport
Vesicle trafficking
Other
B
Shortgrafted vs Homografted
Number of genes
0
20
40
60
Pathway
Cell cycle
Cell wall
Cellular respiration
Chromatin organisation
External stimuli response
Lipid metabolism
Nutrient uptake
Photosynthesis
Phytohormones
Protein metabolism
RNA metabolism
Solute transport
Other
